# Transillumination‐Aided Infiltration of MIH‐Affected Molars: Evaluation of the Clinical Workflow

**DOI:** 10.1111/ipd.70064

**Published:** 2025-12-29

**Authors:** Omar Marouane, Mariem Nefzaoui, David John Manton, Marcus Cebula, Falk Schwendicke, Susanne Effenberger

**Affiliations:** ^1^ Oralys Dental Clinic Tunis Tunisia; ^2^ Pediatric Dentistry Department, La Rabta Hospital Tunis Tunisia; ^3^ Faculty of Dental Medicine of Monastir Monastir Tunisia; ^4^ University of Groningen UMCG, Center for Dentistry and Oral Hygiene Groningen the Netherlands; ^5^ Academic Center for Dentistry Amsterdam (ACTA) University of Amsterdam and Vrije Universiteit Amsterdam Amsterdam the Netherlands; ^6^ Clinical Research, Dental‐Material Gesellschaft mbH Hamburg Germany; ^7^ Department of Conservative Dentistry, Periodontology and Digital Dentistry LMU Klinikum LMU Munich Germany

**Keywords:** developmental defects of enamel, molar incisor hypomineralisation, resin infiltration, transillumination

## Abstract

**Background:**

Enamel affected by molar incisor hypomineralisation (MIH) exhibits reduced mechanical properties due to its disorganised prismatic structure, lower mineral density and higher protein content. These alterations increase porosity and susceptibility to posteruptive enamel breakdown. Minimally invasive treatments, such as resin infiltration, have been proposed to reinforce enamel integrity. Recent advances suggest that transillumination can guide infiltration more accurately, allowing better visualisation of lesion extent and potentially improving treatment outcomes.

**Aim:**

This case series evaluated the clinical suitability of transillumination‐aided resin infiltration, specifically the utility of using transillumination to support resin infiltration treatment, for MIH‐affected molars.

**Design:**

Five MIH‐affected first permanent molars from five paediatric patients, diagnosed according to EAPD criteria for MIH, were included. One molar presented with two distinct lesions, leading to the inclusion of six lesions in total. Transillumination was used to assess the lesion appearance, to assist with selective surface removal and to monitor the resin infiltration process to assess when it was completed, that is, when the lesion either fully disappeared visually or no change was visible anymore under transmitted light.

**Results:**

Transillumination was employed successfully to assist with resin infiltration treatment and to assess the treatment outcome. Four lesions showed partial and two complete infiltrations visually, indicating putatively that the porous lesion structure is strengthened. No complications were reported during treatment.

**Conclusions:**

Transillumination was successfully utilised to support resin infiltration in MIH‐affected molars and to visualise the quality of infiltration, serving as a potential surrogate outcome measure. Future research should explore long‐term clinical outcomes and the impact on patients' quality of life.

## Why this paper is important to pediatric dentists

- Transillumination‐aided infiltration provides a minimally invasive treatment option for MIH‐affected FPMs, with transillumination enhancing lesion detection, guiding precise surface preparation and improving monitoring of resin penetration.
- Even partial infiltration can reinforce the infiltrated hypomineralised enamel, helping to stabilise that area, reduce the risk of PEB and caries lesion development and improve bonding performance for subsequent restorative procedures.
- Resin infiltration offers immediate structural reinforcement and functional benefits, while remaining compatible with adjunctive therapies when indicated.

## 1 Introduction

Molar‐incisor hypomineralisation (MIH) is defined as a developmental enamel defect affecting one to four first permanent molars (FPMs) in varying degrees of severity, with frequent involvement of permanent incisors [1]. The condition presents as well‐demarcated enamel opacities ranging from white‐creamy to yellow‐brown in colour. The global prevalence of MIH is estimated to be around 13.5% [2, 3]. Despite its clinical significance, the precise aetiology of MIH remains unclear, with genetic, epigenetic and systemic factors being implicated [4].

MIH‐affected enamel is associated with a significant reduction in enamel mineral density, hardness, fracture resistance and increased enamel porosity [5]. Consequently, the risk of posteruptive breakdown (PEB), especially in the posterior region where there is increased biomechanical stress, caries lesion development and hypersensitivity, is increased markedly. The latter can influence oral hygiene habits, further increasing the risk of caries lesion development and PEB, and altering overall dental care‐seeking behaviour [6, 7]. Dental treatment of MIH‐affected FPMs is required nearly 10 times more often than for FPMs without MIH [8, 9]. This can contribute to dental anxiety and avoidance behaviours, which may further worsen oral health and negatively impact children's oral health‐related quality of life (OHRQoL), underscoring the importance of effective intervention strategies [5, 6, 9, 10].

Treatment of MIH‐affected FPMs ranges from noninvasive to restorative or even extraction, depending on lesion severity and extent. Caries and PEB preventive strategies include dietary advice, oral hygiene instructions and remineralisation using fluoride‐based or CPP‐ACP‐based products, which have shown potential in strengthening enamel and alleviating hypersensitivity; however, the effectiveness of these approaches may be limited by patient compliance and ongoing caries risk. Another option recommended for early management is resin‐based sealants that aim to physically protect the hypomineralised enamel and create diffusion barriers to block acid penetration and inhibit caries lesion development. Whilst sealants are effective and less reliant on patient cooperation, their retention may be compromised by the decreased bond strength to the hypomineralised enamel [11].

Resin infiltration bridges preventive and restorative care by strengthening weakened enamel [12]. This technique involves the penetration of a low viscosity resin into the porous structure of hypomineralised enamel, thereby reinforcing and stabilising the structure, potentially preventing PEB [12]. The resin‐infiltrated structure, furthermore, serves as an internal diffusion barrier, making the hypomineralised enamel more resistant to the carious process that could develop on the lesion. Unlike sealants, this technique is less reliant on etch/bond retention for its effectiveness [13].

Resin infiltration has been used effectively to treat MIH‐affected anterior teeth [14, 15, 16, 17], primarily to mask enamel opacities; however, its application in molars has different clinical objectives: preventing PEB, reducing caries risk and alleviating hypersensitivity. While recent studies have shown that resin infiltration in MIH‐affected molars significantly decreases the risk of PEB and reduces hypersensitivity, its use and outcomes remain relatively underexplored [12, 18, 19].

To effectively treat MIH‐affected lesions using resin infiltration, modified treatment protocols have been developed and successfully implemented in recent years, including the transillumination‐aided infiltration approach [16, 20].

Transillumination enhances the visibility of hypomineralised regions, aiding in the assessment of lesion characteristics, guiding surface removal by clarifying the appearance of the lesions' edges and monitoring the infiltration progression to determine when it is complete. This technique promotes more effective and more predictable infiltration into the enamel microstructures, thereby improving the overall treatment outcomes [16, 21, 22, 23].

The aim of this case series is to evaluate the use of the transillumination‐aided infiltration approach for the treatment of MIH‐affected molars—a clinically challenging area due to more complex tooth morphology—highlighting the specific considerations, differences in application and distinct clinical objectives compared to its use in anterior teeth.

## 2 Material and Methods

### 2.1 Case Description and Selection

Five patients aged between 13 and 18 years with MIH‐affected FPMs were recruited, representing a convenience sample. Diagnosis was established by clinical examination according to the European Academy of Paediatric Dentistry (EAPD) criteria. For treatment, six individual lesions from five FPMs—one molar per patient—were included, with lesion colour ranging from white‐creamy to yellow‐brown. Some lesions presented with PEB, but no associated caries lesions were present. No hypersensitivity was reported by any of the patients in relation to the included FPMs. Clinical characteristics are summarised in Table 1.

**TABLE 1:** Lesion characteristics and treatment outcomes observed after infiltration.

Case/lesion number*	Discoloration	Substance loss	Resin restoration	Infiltration quality	Location
1	No	No	No	Complete	Buccal
2	Yes	Yes	No	Partial	Buccal
3	Yes	No	No	Partial	Buccal
4	Yes	Yes	Yes	Partial	Occlusal and buccal
5	Yes	No	No	Partial	Occlusal
6	Yes	No	No	Complete	Buccal

* Cases/lesions five and six refer to two distinct lesions located on the same MIH‐affected molar, while cases one through four correspond to four individual lesions from four separate MIH‐affected molars. Only one MIH‐affected molar per patient was included in the study.

### 2.2 Ethical Considerations

Ethical approval for this case series was given by the local institutional board of Farhat Hached Hospital, Sousse, Tunisia (12/2019, IRB:8931). Recruitment was initiated in March 2025 and completed in August 2025. Participants were recruited during routine clinical examination from those presenting at the clinic. Participants were informed and understood that the success of the treatment is difficult to predict, potentially varying between complete visual disappearance of the lesions and partial aesthetic improvement.

### 2.3 Resin Infiltration Protocol

**FIGURE 1:**
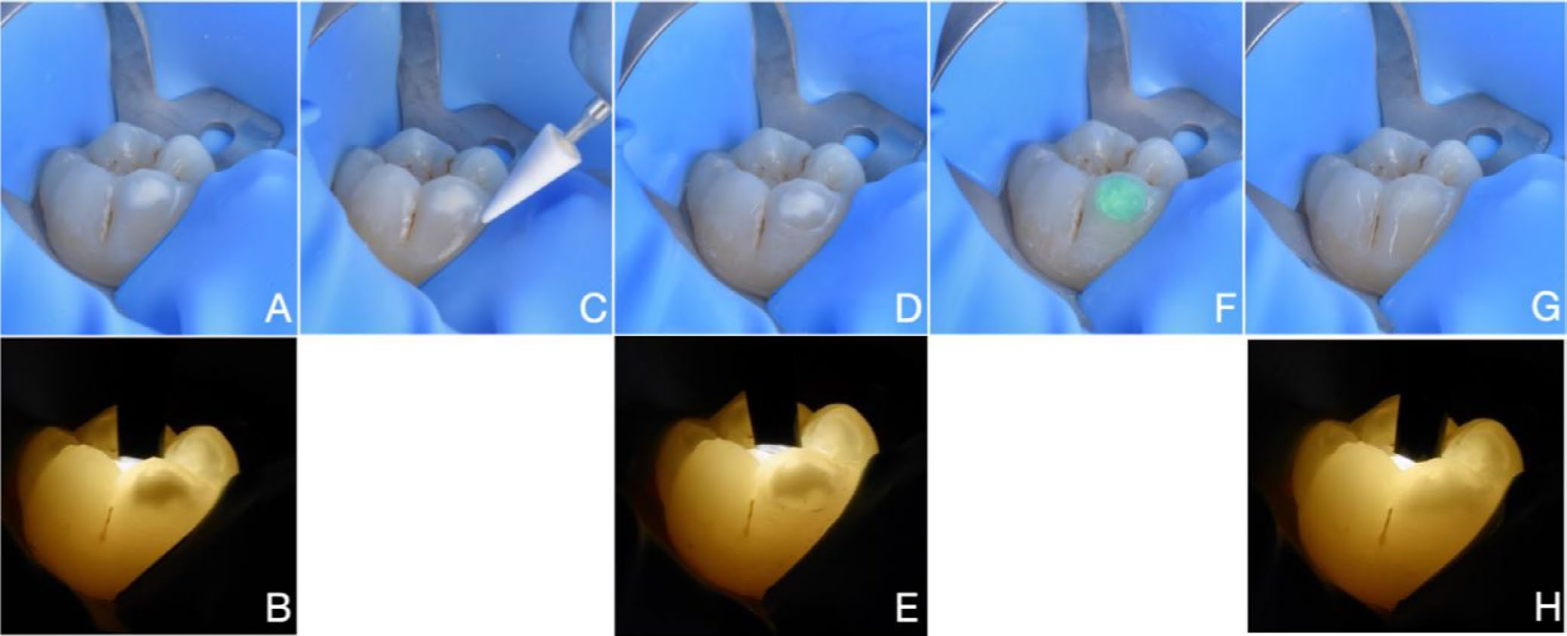
CASE 1: (A, B) Preoperative views of a white MIH lesion affecting the buccal surface of the lower first molar, shown under reflected and transmitted light. (C) Surface layer removal was performed using an aluminium oxide finishing abrasive stone. (D, E) Appearance of the lesion after surface layer removal under reflected and transmitted light. Well‐defined margins were clearly visible under transillumination. (F) The exposed hypomineralised enamel was etched during 120 s with 15% HCl (Icon Etch, DMG); (G, H) Immediate postoperative view of the lesion after complete infiltration under reflected and transmitted light. The masking of the lesion indicates successful infiltration.

**FIGURE 2:**
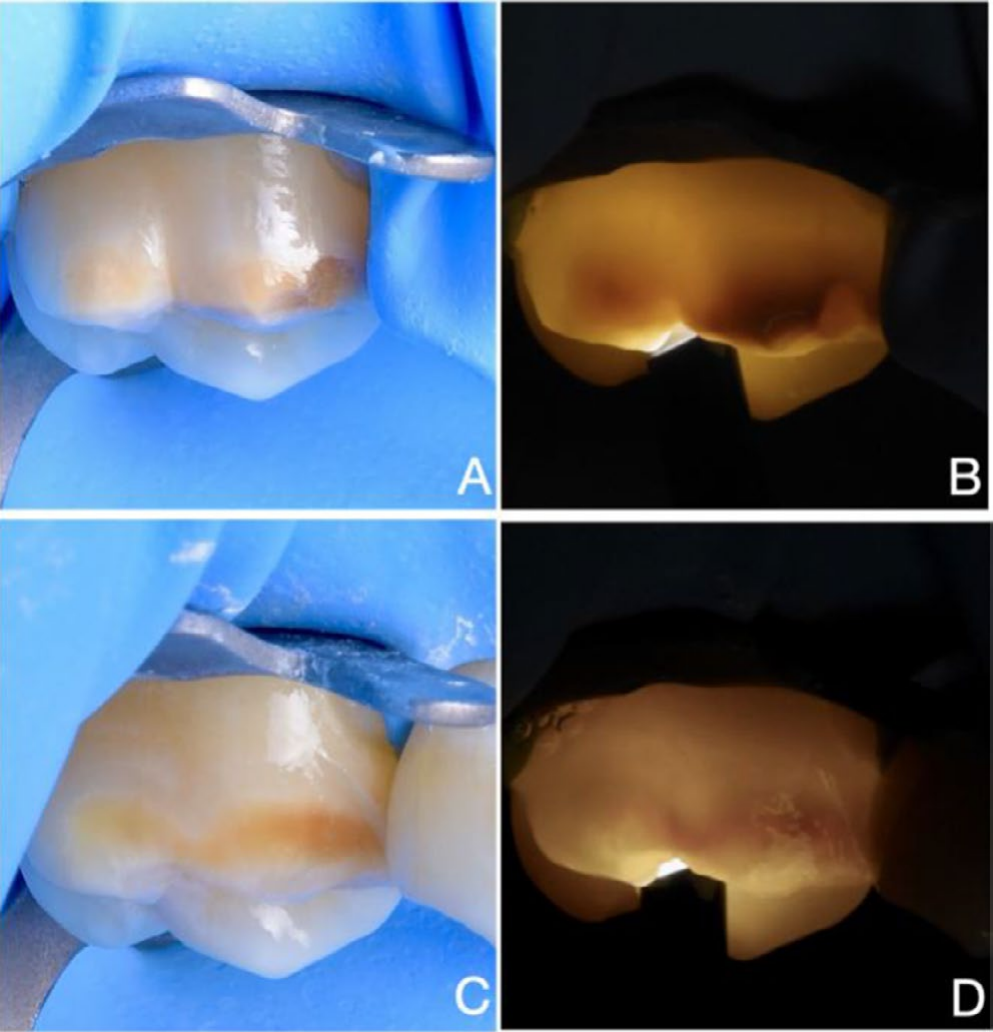
CASE 2: (A, B) Preoperative and (C, D) immediate postoperative views of a yellow‐brown MIH lesion affecting the buccal surface of the upper first molar with minimal PEB, shown under transmitted and reflected light. Areas that remain dark after treatment indicate partial infiltration, while areas that appear masked indicate complete infiltration. Following the resin infiltration treatment, composite restoration of the pre‐existing enamel loss was not required.

**FIGURE 3:**
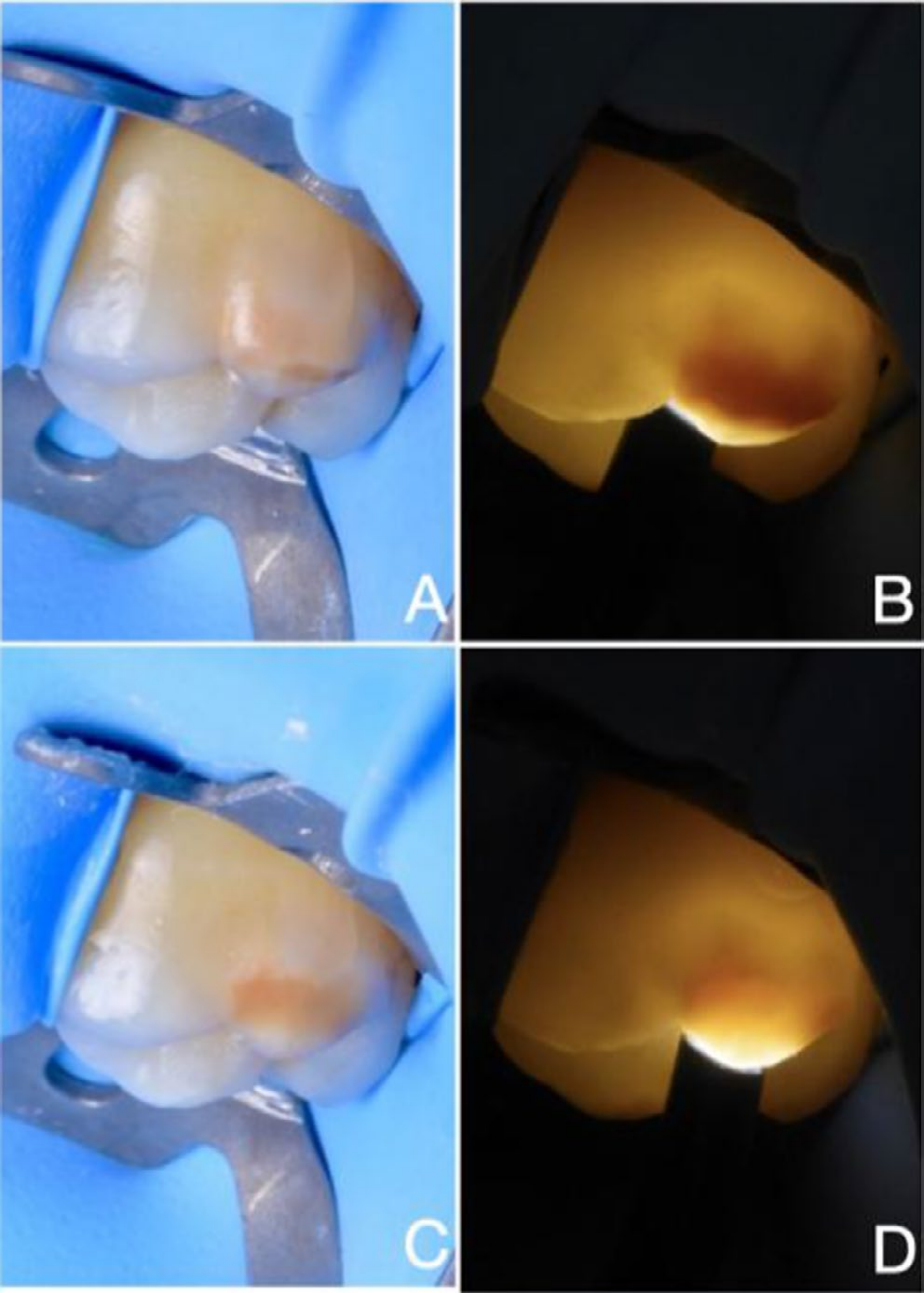
CASE 3: (A, B) Preoperative and (C, D) immediate postoperative views of a yellow‐brown MIH lesion affecting the buccal surface of the upper first molar, shown under transmitted and reflected light. Areas that remain dark after treatment indicate partial infiltration, while areas that appear masked indicate complete infiltration.

**FIGURE 4:**
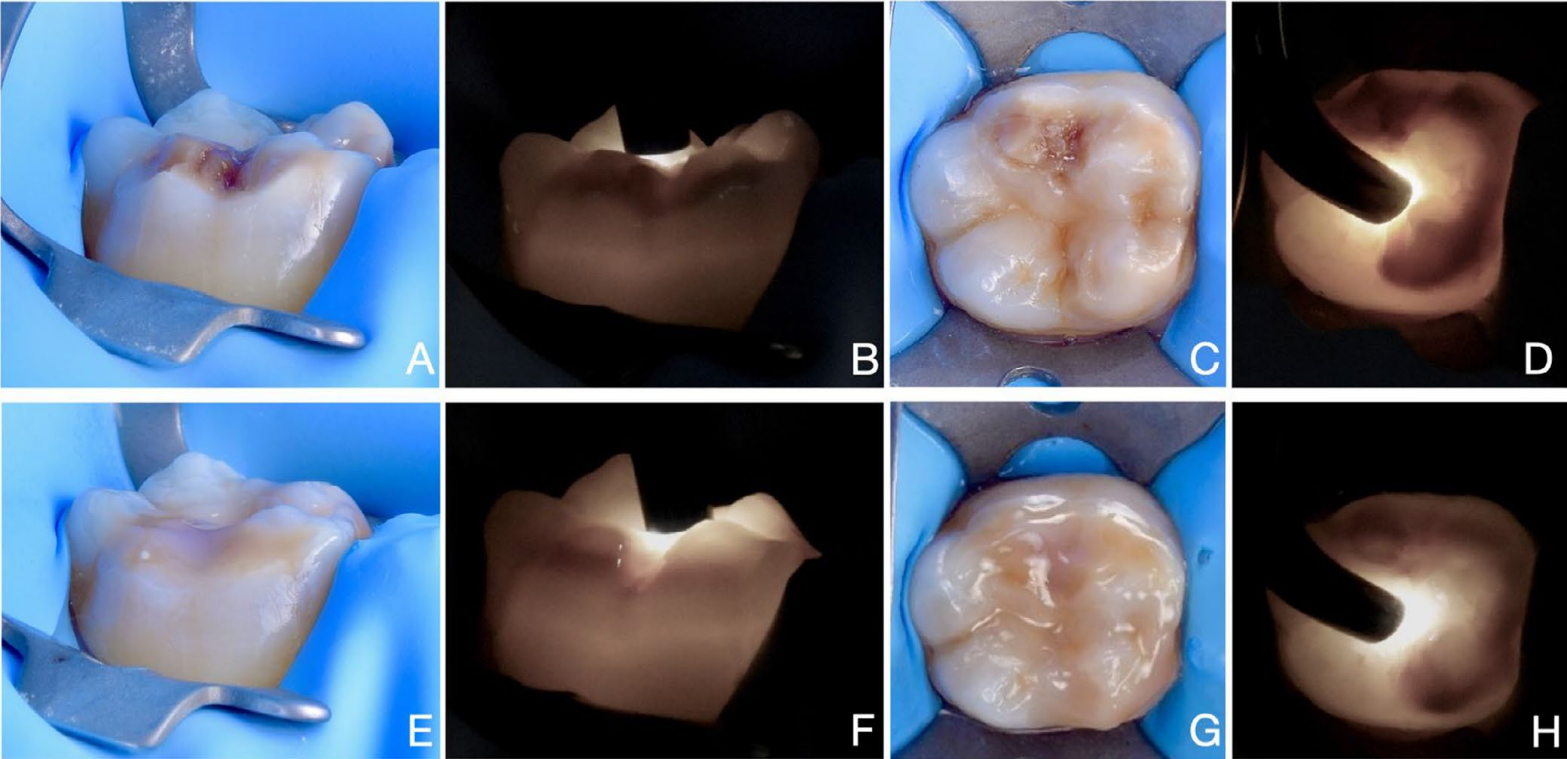
CASE 4: Preoperative buccal (A, B) and occlusal (C, D) views as well as immediate postoperative (after resin infiltration) buccal (E, F) and occlusal (G, H) views of a yellow‐brown MIH lesion with PEB affecting the lower first molar, shown under transmitted and reflected light. Areas that remain dark after treatment indicate partial infiltration, while areas that appear masked indicate complete infiltration. The infiltrated lesion was subsequently covered with composite to restore the pre‐existing enamel substance loss.

**FIGURE 5 and 6:**
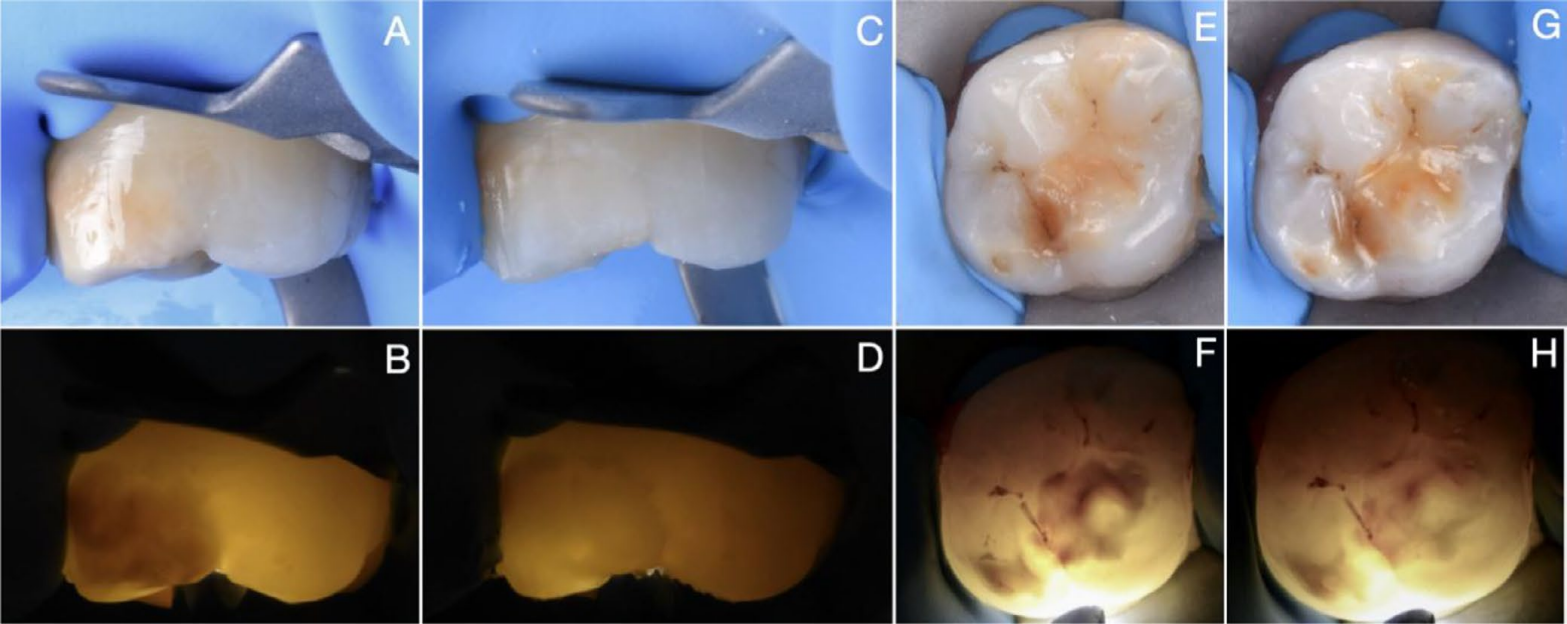
CASE 5 and 6: (A, B, E, F) Preoperative and (C, D, G, H) immediate postoperative views of two MIH lesions affecting both the buccal and occlusal surfaces of the upper first molar, shown under transmitted and reflected light. These cases illustrate that MIH detection and treatment can also be effectively performed on the occlusal surface. Areas that remain dark after treatment indicate partial infiltration, while areas that appear masked indicate successful infiltration.

To prevent further structural deterioration and to reduce the risk of caries lesion development, the hypomineralised lesions on all affected molars were treated with resin infiltration (Icon; DMG, Hamburg, Germany) by the same experienced clinician (O.M.), following the transillumination‐aided infiltration protocol [20, 22]. This involved the gentle removal of the pseudo‐intact surface layer covering the lesion until well‐defined margins at the sound enamel‐lesion interface became clearly visible under transmitted light, indicating that the lesion body has been exposed—an essential prerequisite for effective resin infiltration. Without local anaesthesia, the surface layer removal was performed using an aluminium oxide finishing abrasive stone (Cerastone; EVE Ernst Vetter, Pforzheim, Germany) in a high‐speed handpiece with water spray (Figure 1C), using rubber dam isolation. The procedure was repeated as necessary until well‐defined margins were clearly visible under transillumination (Figure 1B,E). To monitor the process, a handheld LED transilluminator with a 3 mm glass light guide (Microlux Transilluminator; AdDent, CT, USA) was positioned perpendicular to the occlusal (Figures 1, 2, 3, 4, 5 and 6) or the buccal (Figure 6) surface.

Once the surface layer was removed adequately, the remaining hypomineralised enamel was etched with 15% hydrochloric acid (Icon Etch; DMG) for 120 s, in accordance with the manufacturer's instructions (Figure 1F). The surface was then thoroughly rinsed for 30 s using a triplex water spray. Ethanol (Icon Dry; DMG) was applied for 30 s to desiccate the lesion.

The lesion was then infiltrated using a low‐viscosity resin (Icon Infiltrant; DMG), which was applied with a disposable micro‐applicator brush in a circular motion until the opacity disappeared or no further progress was visible. Transillumination was used to monitor the infiltration process in real time, allowing precise visualisation of resin penetration during application and enabling a reliable assessment of when the infiltration process was complete. The resin was then light‐cured (MiniLED active; ACTEON, Bordeaux, France) for 40 s. Following the manufacturer's recommendation, a second application of resin was placed for 60 s and light‐cured again for 40 s. One case (Figure 4) required subsequent resin composite (Ecosite One; DMG) placement to restore pre‐existing enamel loss.

### 2.4 Outcome Measures

The main goal of infiltrating MIH lesions in molars is to reduce PEB and the risk of caries by stabilising the lesions and creating a diffusion barrier, effects that can only be fully assessed in the long term. In this case series, short‐term success was evaluated by the same clinician who performed the treatment using a trichotomous assessment (no infiltration/partial infiltration/complete infiltration) based on lesion appearance under transillumination, which provides enhanced visualisation of the extent and quality of infiltration. Secondary outcomes included patient‐reported hypersensitivity and treatment‐related adverse events.

## 3 Results

Among the six cases presented, four showed partial infiltration under both reflected light and transillumination (Table 1 and Figures 2, 3, 4, 5 and 6), whilst two showed complete infiltration under transillumination and reflected light (Table 1 and Figures 1 and 6). None of the cases exhibited hypersensitivity before, during or after treatment, and no other adverse effects were noted.

## 4 Discussion

In this case series, the application of the transillumination‐aided infiltration concept for managing MIH‐affected molars is described, encompassing the different clinical forms of MIH lesions—from mild opacities to more extensive hypomineralised breakdown—that can be addressed through this minimally invasive treatment approach. Given that resin infiltration remains a technique‐sensitive and sometimes unpredictable procedure for managing MIH lesions, the rationale behind this concept is to establish a standardised clinical protocol. By incorporating transillumination into the resin infiltration treatment of MIH‐affected molars, the aim is to optimise detection of lesion extension, guide precise enamel surface preparation to minimise tissue removal and monitor infiltration progression—ultimately improving treatment outcomes and reproducibility.

In case of anterior teeth, light is typically transmitted from the palatal or lingual surface to illuminate labial lesions effectively [21, 22]. Detecting labial or buccal and occlusal lesions in molars, however, often requires light transmission from the occlusal surface (Figures 1, 2, 3, 4, 5 and 6) or buccal surface (Figure 6). Furthermore, access in the posterior region is more limited due to anatomical constraints, and controlling moisture is significantly more difficult. For these reasons, rubber dam isolation is essential—particularly for mandibular molars—to ensure a dry and accessible working field.

MIH lesions often present with discolouration due to elevated levels of organic material, making it difficult to monitor the resin infiltration process and assess its success under reflected light. In fact, increased discolouration is frequently observed after treatment, as seen in Figures 2, 3 and 5. This effect can be explained by the reduction in light scattering within the porous enamel matrix after infiltration, which permits greater light penetration and interaction with subsurface proteins and chromophores. The resulting increase in light absorption intensifies the perceived discoloration. While this chromatic enhancement may serve as a visual indicator showing that the lesion's porous architecture has been thoroughly infiltrated and thus structurally stabilised, it remains challenging to interpret. In contrast, transillumination provides a higher contrast, allowing more accurate monitoring of the infiltration process, thus preventing premature curing and better evaluation of the final result.

Given the increased biomechanical stress in the posterior area, the goal of resin infiltration is rather to improve the mechanical properties of the MIH‐lesion. This is an important factor for successful restorative treatment and for preventing PEB in the first place. It is postulated that infiltration may reduce porosity and permeability while increasing the hardness of the lesion [24, 25, 26]. This, in turn, may help prevent or reduce PEB and caries lesion development and improve the bond strength of a resin‐based restoration at the infiltrated surface [27, 28]. Improved bonding minimises the risk of marginal breakdown and therefore the likelihood of treatment failure. Considering these objectives, even partial infiltration (as seen in Figures 2, 3, 4, 5 and 6) may be considered a success in molars, as the enamel is reinforced and as aesthetics are of lesser concern than in the case of anterior teeth, where complete infiltration (and sometimes bleaching) is required for full masking to meet aesthetic demands. Despite the growing interest in resin infiltration for MIH‐affected molars, the literature on this subject remains sparse [25, 29]. More studies are needed to better understand the long‐term efficacy of this approach.

While resin infiltration offers many advantages, it is not a viable treatment for all cases. The cases presented here were well‐suited due to the absence of extensive PEB and sensitivity, complete molar eruption and cooperative patients. When MIH lesions exhibit extensive PEB, conventional restorative treatment is generally recommended. This involves the removal of the remaining hypomineralised enamel and placement of a definitive restoration. Furthermore, if effective rubber dam isolation cannot be ensured, alternative treatment approaches should be considered in place of resin infiltration, such as remineralisation and sealing, each having distinct advantages and limitations.

Remineralisation using regular topical application of fluoride or CPP‐ACP containing products can reduce tooth sensitivity, enhance resistance to demineralisation and increase surface microhardness [30, 31, 32, 33]. However, their reliance on prolonged and consistent application makes them less practical as the effectiveness heavily relies on patient compliance [34]. Moreover, unlike resin infiltration, which provides immediate structural reinforcement, remineralising agents require more time to produce a therapeutic effect [35].

Sealing protects MIH‐affected tissue from biofilm acids and enamel breakdown by creating a superficial barrier. This technique does not rely on ongoing patient compliance and provides an immediate protective effect. However, resin‐based sealants exhibit significantly lower retention rates on MIH lesions compared to healthy enamel, leading to a heightened risk of early failure and the need for retreatment. Retention challenges are particularly pronounced when hypomineralisation directly impacts the pits and fissures, areas where strong bonding is crucial [11, 35, 36]. Additionally, sealants require periodic monitoring and maintenance to ensure their continued effectiveness. In contrast to sealants, resin infiltration penetrates deeper and reinforces the enamel structure, providing longer‐lasting protection and potential desensitisation benefits. Although sealants are easier to apply, the superior micromechanical integration of resin infiltration may make it a more effective approach or adjunctive option in severe MIH cases. Additionally, unlike sealants, resin infiltration is suitable for treating smooth surfaces.

Taking a broader perspective, prior resin infiltration could enhance the longevity of sealed fissures by promoting strong bonding. Moreover, these two methods can act additively as they have different objectives—infiltration targets the lesion, whilst the sealant protects the external surface, limits plaque accumulation and reduces the risk of caries lesion development.

If patient cooperation is limited or proper isolation cannot be achieved, limiting caries risk, remineralisation strategies or sealants may be more appropriate. Otherwise, resin infiltration should be prioritised as the initial approach, whether combined with other therapeutic strategies or not. This strategy aims to optimise hypomineralisation lesion stabilisation whilst ensuring a minimally invasive approach, thereby minimising the need for more invasive restorative interventions in the long term.

This case series illustrates the potential application of transillumination‐aided resin infiltration for MIH‐affected molars. Given the functional importance of FPMs and their exposure to mechanical stress, this technique may help enhance the structural integrity of lesions. While the findings are based on only six lesions and should be interpreted cautiously, transillumination‐aided infiltration offers a minimally invasive, function‐preserving approach that could reduce the need for repeated restorative procedures. Further studies with larger cohorts and appropriate follow‐up periods are needed to confirm these preliminary observations and fully establish clinical relevance.

## Author Contributions

Omar Marouane and Mariem Nefzaoui: conceived the concept of the study; Omar Marouane, Mariem Nefzaoui and Susanne Effenberger: devised the methodology; Falk Schwendicke, Marcus Cebula and David John Manton: performed data validation and analysis; Omar Marouane: clinical treatments and visualisation of cases; David John Manton and Falk Schwendicke: project supervision; Susanne Effenberger: project administration; Mariem Nefzaoui and Omar Marouane: writing original draft; Marcus Cebula, Falk Schwendicke and David John Manton: writing – review and editing of the final manuscript. All authors have read and agreed to the submitted version of the work.

## Funding

The authors have nothing to report.

## Ethics Statement

Ethical approval for this observational study was given by the local institutional board of Farhat Hached Hospital, Sousse, Tunisia (12/2019, IRB:8931).

## Conflicts of Interest

Omar Marouane and Mariem Nefzaoui do not have a conflicts of interest. Marcus Cebula and Susanne Effenberger are employees of DMG Dental‐Material Gesellschaft mbH, but do not receive any personal benefits from the sales of products used in this study. Falk Schwendicke and David John Manton are consultants and give lectures for DMG Dental‐Material Gesellschaft mbH, but do not receive any personal benefits from the sales of products used in this study. Susanne Effenberger is a part‐time employee of the department headed by Falk Schwendicke.

## Data Availability

The data that support the findings of this study are available from the corresponding author upon reasonable request.

## References

[1] K. L. Weerheijm , B. Jalevik , and S. Alaluusua , “Molar‐Incisor Hypomineralisation,” Caries Research 35, no. 5 (2001): 390, 10.1159/000047479. 11641576

[2] F. Schwendicke , K. Elhennawy , S. Reda , K. Bekes , D. J. Manton , and J. Krois , “Global Burden of Molar Incisor Hypomineralization,” Journal of Dentistry 68 (2018): 10–18, 10.1016/j.jdent.2017.12.002. 29221956

[3] F. Schwendicke , K. Elhennawy , S. Reda , K. Bekes , D. J. Manton , and J. Krois , “Corrigendum to “sisor Hypomineralization” [J. Dent. 68C (2018) 10‐18],” Journal of Dentistry 80 (2019): 89–92, 10.1016/j.jdent.2018.11.006. 30554832

[4] F. Crombie , D. Manton , and N. Kilpatrick , “Aetiology of Molar‐Incisor Hypomineralization: A Critical Review,” International Journal of Paediatric Dentistry 19, no. 2 (2009): 73–83, 10.1111/j.1365-263X.2008.00966.x. 19250392

[5] K. Elhennawy , D. J. Manton , F. Crombie , et al., “Structural, Mechanical and Chemical Evaluation of Molar‐Incisor Hypomineralization‐Affected Enamel: A Systematic Review,” Archives of Oral Biology 83 (2017): 272–281, 10.1016/j.archoralbio.2017.08.008. 28843745

[6] S. Shields , T. Chen , F. Crombie , D. J. Manton , and M. Silva , “The Impact of Molar Incisor Hypomineralisation on Children and Adolescents: A Narrative Review,” Healthcare (Basel) 12, no. 3 (2024): 30370, 10.3390/healthcare12030370. PMC10855782 38338255

[7] K. R. Weber , R. J. Wierichs , H. Meyer‐Lueckel , and S. Flury , “Restoration of Teeth Affected by Molar‐Incisor Hypomineralisation: A Systematic Review,” Swiss Dental Journal 131, no. 12 (2021): 988–997, 10.61872/sdj-2021-12-764. 33764037

[8] Z. Al‐Nerabieah , M. AlKhouli , and M. Dashash , “Navigating the Complexities of Molar Incisor Hypomineralization: Challenges and Strategies in Pediatric Dentistry,” International Journal of Dentistry 2025 (2025): 9329492, 10.1155/ijod/9329492. 39811496 PMC11732276

[9] B. Jalevik , N. Sabel , and A. Robertson , “Can Molar Incisor Hypomineralization Cause Dental Fear and Anxiety or Influence the Oral Health‐Related Quality of Life in Children and Adolescents?‐A Systematic Review,” European Archives of Paediatric Dentistry 23, no. 1 (2022): 65–78, 10.1007/s40368-021-00631-4. 34110616 PMC8927003

[10] J. L. Goncalves , F. K. Carvalho , A. M. Queiroz , and F. W. G. Paula‐Silva , “Implications of Histological and Ultrastructural Characteristics on the Chemical and Mechanical Properties of Hypomineralised Enamel and Clinical Consequences,” Monographs in Oral Science 32 (2024): 43–55, 10.1159/000538865. 39321779

[11] N. A. Lygidakis , E. Garot , C. Somani , G. D. Taylor , P. Rouas , and F. S. L. Wong , “Best Clinical Practice Guidance for Clinicians Dealing With Children Presenting With Molar‐Incisor‐Hypomineralisation (MIH): An Updated European Academy of Paediatric Dentistry Policy Document,” European Archives of Paediatric Dentistry 23, no. 1 (2022): 3–21, 10.1007/s40368-021-00668-5. 34669177 PMC8926988

[12] V. K. C. Nogueira , I. P. Mendes Soares , C. M. B. Fragelli , et al., “Structural Integrity of MIH‐Affected Teeth After Treatment With Fluoride Varnish or Resin Infiltration: An 18‐Month Randomized Clinical Trial,” Journal of Dentistry 105 (2021): 103570, 10.1016/j.jdent.2020.103570. 33385533

[13] S. Paris , H. Meyer‐Lueckel , H. Colfen , and A. M. Kielbassa , “Resin Infiltration of Artificial Enamel Caries Lesions With Experimental Light Curing Resins,” Dental Materials Journal 26, no. 4 (2007): 582–588, 10.4012/dmj.26.582. 17886464

[14] G. D. S. Athayde , P. Reis , R. C. Jorge , G. C. A. Americano , T. Fidalgo , and V. M. Soviero , “Impact of Masking Hypomineralization Opacities in Anterior Teeth on the Esthetic Perception of Children and Parents: A Randomized Controlled Clinical Trial,” Journal of Dentistry 123 (2022): 104168, 10.1016/j.jdent.2022.104168. 35643218

[15] N. Q. Hoan , N. P. Huyen , D. C. Son , D. H. Thien , C. J. Sabet , and V. T. N. Ngoc , “Effectiveness of Resin Infiltration in the Management of Anterior Teeth Affected by Molar Incisor Hypomineralisation (MIH): A Systematic Review and Meta‐Analysis,” Journal of Dentistry 149 (2024): 105254, 10.1016/j.jdent.2024.105254. 39067648

[16] O. Marouane and D. J. Manton , “The Influence of Lesion Characteristics on Application Time of an Infiltrate Applied to MIH Lesions on Anterior Teeth: An Exploratory In Vivo Pilot Study,” Journal of Dentistry 115 (2021): 103814, 10.1016/j.jdent.2021.103814. 34543698

[17] N. A. Prado , R. C. Jorge , R. F. Moreira , et al., “Does the Application Protocol Influence the Masking Effect of Resin Infiltration on MIH Opacities? Systematic Review and Meta‐Analysis,” Journal of Dentistry 155 (2025): 105617, 10.1016/j.jdent.2025.105617. 39947580

[18] L. Giannetti , A. Murri Dello Diago , G. Silingardi , and E. Spinas , “Superficial Infiltration to Treat White Hypomineralized Defects of Enamel: Clinical Trial With 12‐Month Follow‐Up,” Journal of Biological Regulators and Homeostatic Agents 32, no. 5 (2018): 1335–1338. 30334435

[19] V. Luppieri , D. Porrelli , L. Ronfani , G. Turco , and M. Cadenaro , “A Resin Infiltration Technique for Molar Hypomineralization Treatment: A Preliminary Study in a Pediatric Population,” Pediatric Dentistry 44, no. 5 (2022): 322–325. 36309779

[20] O. Marouane and F. Chtioui , “Transillumination‐Aided Infiltration: A Diagnostic Concept for Treating Enamel Opacities,” Journal of Esthetic and Restorative Dentistry 32, no. 5 (2020): 451–456, 10.1111/jerd.12602. 32497384

[21] O. Marouane and N. Douki , “The Use of Transillumination in Detecting Subclinical Extensions of Enamel Opacities,” Journal of Esthetic and Restorative Dentistry 31, no. 6 (2019): 595–600, 10.1111/jerd.12506. 31215759

[22] O. Marouane , D. J. Manton , M. Cebula , F. Schwendicke , and S. Effenberger , “In Vivo Comparison of Resin Infiltration Outcomes Under Different Light Conditions: A Randomized Controlled Clinical Trial,” Journal of Dentistry 153 (2025): 105554, 10.1016/j.jdent.2024.105554. 39746437

[23] C. R. G. Torres , T. P. Pereira , S. Effenberger , and A. B. Borges , “Optimizing Resin Infiltration Procedure in Molar Incisor Hypomineralization Lesions,” Journal of Esthetic and Restorative Dentistry 37, no. 4 (2025): 827–833, 10.1111/jerd.13358. 39474718 PMC12080095

[24] F. Crombie , D. Manton , J. Palamara , and E. Reynolds , “Resin Infiltration of Developmentally Hypomineralised Enamel,” International Journal of Paediatric Dentistry 24, no. 1 (2014): 51–55, 10.1111/ipd.12025. 23410530

[25] M. Soveral , V. Machado , J. Botelho , J. J. Mendes , and C. Manso , “Effect of Resin Infiltration on Enamel: A Systematic Review and Meta‐Analysis,” Journal of Functional Biomaterials 12, no. 3 (2021): 30048, 10.3390/jfb12030048. PMC8395859 34449679

[26] B. Tseveenjav , A. Mulic , J. Waltimo‐Siren , and A. Tulek , “Penetration Depth and Enamel Hardness Effects of Resin Infiltrate and Fissure Sealant in MIH‐Affected Molars: An In‐Vitro Comparison,” European Archives of Paediatric Dentistry (2025): 1–10, 10.1007/s40368-025-01122-6. 41108487

[27] J. He , L. Chen , J. Tian , et al., “Effect of NaOCl or EDTA Pretreatments on the Shear Bond Strength of the Resin‐Infiltrated White Spot Lesions to the Resin Composite: An In Vitro Study,” BMC Oral Health 25, no. 1 (2025): 1494, 10.1186/s12903-025-06900-8. 41023953 PMC12482125

[28] C. Solanke , H. Shokoohi‐Tabrizi , A. Schedle , and K. Bekes , “Shear Bond Strengths of Composite Resin Bonded to MIH‐Affected Hard Tissues With Different Adhesives and Pre‐Treatments,” Dentistry Journal 13, no. 8 (2025): 377, 10.3390/dj13080377. 40863080 PMC12385336

[29] H. Kumar , J. E. A. Palamara , M. F. Burrow , and D. J. Manton , “An Investigation Into the Effect of a Resin Infiltrant on the Micromechanical Properties of Hypomineralised Enamel,” International Journal of Paediatric Dentistry 27, no. 5 (2017): 399–411, 10.1111/ipd.12272. 27813257

[30] I. Cardoso‐Martins , S. Arantes‐Oliveira , A. Coelho , S. Pessanha , and P. F. Marques , “Evaluation of the Efficacy of CPP‐ACP Remineralizing Mousse in MIH White and Yellow Opacities‐In Vitro Vickers Microhardness Analysis,” Dent J (Basel) 10, no. 10 (2022): 186, 10.3390/dj10100186. 36285996 PMC9600031

[31] M. Pasini , M. R. Giuca , M. Scatena , R. Gatto , and S. Caruso , “Molar Incisor Hypomineralization Treatment With Casein Phosphopeptide and Amorphous Calcium Phosphate in Children,” Minerva Stomatologica 67, no. 1 (2018): 20–25, 10.23736/S0026-4970.17.04086-9. 28975773

[32] E. C. Reynolds , F. Cai , N. J. Cochrane , et al., “Fluoride and Casein Phosphopeptide‐Amorphous Calcium Phosphate,” Journal of Dental Research 87, no. 4 (2008): 344–348, 10.1177/154405910808700420. 18362316

[33] V. William , L. B. Messer , and M. F. Burrow , “Molar Incisor Hypomineralization: Review and Recommendations for Clinical Management,” Pediatric Dentistry 28, no. 3 (2006): 224–232. 16805354

[34] C. Baroni and S. Marchionni , “MIH Supplementation Strategies: Prospective Clinical and Laboratory Trial,” Journal of Dental Research 90, no. 3 (2011): 371–376, 10.1177/0022034510388036. 21149856

[35] C. M. B. Fragelli , J. F. Souza , D. G. Bussaneli , F. Jers , L. D. Santos‐Pinto , and R. C. L. Cordeiro , “Survival of Sealants in Molars Affected by Molar‐Incisor Hypomineralization: 18‐Month Follow‐Up,” Brazilian Oral Research 31 (2017): e30, 10.1590/1807-3107BOR-2017.vol31.0030. 28489117

[36] F. Zollner , K. F. Fresen , R. Gaballah , et al., “Effectiveness of Fissure Sealants in 8‐ to 10‐Year‐Olds With and Without Molar‐Incisor Hypomineralization (MIH) ‐ Results From a Cross‐Sectional Epidemiological Study,” Clinical Oral Investigations 29, no. 1 (2024): 20, 10.1007/s00784-024-06083-6. 39692926 PMC11655578

